# Estimating the Optimal Control of Zoonotic Visceral Leishmaniasis by the Use of a Mathematical Model

**DOI:** 10.1155/2013/810380

**Published:** 2013-08-05

**Authors:** Laila Massad Ribas, Vera Lucia Zaher, Helio Junji Shimozako, Eduardo Massad

**Affiliations:** ^1^School of Medicine, University of São Paulo and LIM 01-HCFMUSP, Avenida Dr. Arnaldo 455, 01246-903 São Paulo, SP, Brazil; ^2^London School of Hygiene and Tropical Medicine, University of London, UK

## Abstract

We argue that the strategy of culling infected dogs is not the most efficient way to control zoonotic visceral leishmaniasis (ZVL) and that, in the presence of alternative control strategies with better potential results, official programs of compulsory culling adopted by some countries are inefficient and unethical. We base our arguments on a mathematical model for the study of control strategies against ZVL, which allows the comparison of the efficacies of 5, alternative strategies. We demonstrate that the culling program, previously questioned on both theoretical and practical grounds is the less effective control strategy. In addition, we show that vector control and the use of insecticide-impregnated dog collars are, by far, more efficient at reducing the prevalence of ZVL in humans.

## 1. Introduction

Zoonotic visceral leishmaniasis (ZVL) is one of the most important emerging parasite diseases [1]. It is endemic in rural areas of South America and is caused by *Leishmania infantum* (syn., *L. chagasi*). The parasite is transmitted by the phlebotomine sandfly, *Lutzomyia longipalpis,* and the domestic dog is the main reservoir of the parasite in these areas [2, 3]. Once introduced into a community, the parasite is maintained in a dog-insect-dog peridomestic transmission cycle [1], and occasionally infected flies bite people, causing ZVL. Therefore, the prevalence and the incidence of canine ZVL are important epidemiological parameters for controlling transmission [4], and the estimation of which depends on the reliable identification of infected dogs [5]. As a result, control programs for ZVL often include elimination of infected dogs [6]. Control programs also include detection and treatment of human cases and vector control [7]. More recently, however, two additional control programs have been suggested as alternative or complementarily related to the classical approach, namely, insecticide-impregnated dog collars [8] and a transmission-blocking vaccine [9]. 

In the Mediterranean, where dogs are considered “valuable” [10], ZVL is both a medical and a veterinary problem. In Brazil, the country were 90% of New World's ZVL cases are reported, human cases are numerous and where human cases are many and dogs are considered “less valuable” [10], ZVL is chiefly considered to be only a medical problem. As a result, 850,000 dogs are screened annually in Brazil, and between 20,000 [11] and 25,000 [10] dogs are culled upon positive diagnosis. This perspective of undervaluing dogs is the official position of some governments like the Brazilian Health Authorities, which forbid the treatment of infected dogs with drugs of human use (Interministerial law no. 1,426 of July 11 2008, Editorial of Clinica Veterinária 83: 16-17, 2009) and impose the elimination of seropositive dogs as compulsory. Health agents threaten dog owners with heavy fines for refusing the serological analysis of their animals, and thousands of diagnosed dogs, with a high probability of being false positives, are eliminated every year without a counterproof. Hence, instead of submitting their dogs to the serological screening, owners refuse the entrance of health agents into their homes or transfer their animals to areas where there is no serological screening. Some owners even avoid taking their dogs to the veterinary doctors fearing some examination that could result in a death sentence to their animals [12].

From the ethical point of view, two aspects must be highlighted: the ethics of animals (from the veterinarian point of view) and the ethics of human relations, where those animals and their relationship with the human society are increasingly valued (some authors, like Singer [13], discuss the “importance” of animals on the same parameters as humans) and the human health is at risk. Conflict between these aspects should be avoided whenever possible.

In this paper we argue that the strategy of culling infected dogs is not the most efficient way to control ZVL and that in the presence of alternative control strategies with better potential results the Brazilian official program of compulsory culling is unethical. We base our arguments on a mathematical model for the study of control strategies against ZVL [14], which allows for the comparison between the efficacies of 5 alternative strategies. We demonstrate that the culling program, previously doubted both on theoretical [10] and practical grounds [15, 16], is one of the less effective methods, and vector control and the use of insecticide-impregnated dog collars are, by far, more efficient in reducing the prevalence of ZVL in humans.

## 2. Materials and Methods

We used a mathematical model that is an adaptation of the one proposed by Burattini et al. [14] and assumes a human and a dog population, with the biological vector transmitting the infection within and between the two populations. The three populations (humans, dogs, and vectors) are assumed constants. They are divided into eleven categories that describe the proportions that are human (indexed as *h*) and dog (indexed as *d*), the subpopulations that are susceptible (*x*_*h*_ and *x*_*d*_), infected but without noticeable disease (*l*_*h*_ and *l*_*d*_) (i.e., “latent”), clinically ill (*y*_*h*_ and *y*_*d*_), and recovered immunes (*z*_*h*_ and *z*_*d*_) and the three possible states of the vector population: noninfected, infected but not infective, and infective individuals, denoted as *s*_1_, *s*_2_, and *s*_3_, respectively. The set of differential equations describing the model's dynamics is

(1) dxhdt=(lh+yh+zh)μh+αhyh+rhlh+γhzh−bahxhmhs3,dlhdt=bahxhmhs3−(rh+μh+δh+φh)lh,dyhdt=φhlh−(μh+αh+τh)yh,dzhdt=δhlh+τhyh−(μh+γh)zh,dxddt=(ld+yd+zd)μd+αdyd+rdld+γdzd−b(adθ)xdmds3+ωdyd,dlddt=b(adθ)xdmds3−(rd+μd+δd+φd+ξd)ld,dyddt=φdld−(μd+αd+τd−ωd−ξd)yd,dzddt=δdld+τdyd−(μd+γd)zd,ds1dt=μs(s2+s3)−[(cν)(adθ)(ld+yd)]s1,ds2dt=[(cν)(adθ)(ld+yd)]s1−[(cν)ad(ld(t−τ)+yd(t−τ))]×s1(t−τ)exp⁡(−μsτ)−μss2,ds3dt=[(cν)(adθ)(ld(t−τ)+yd(t−τ))]×s1(t−τ)exp⁡⁡(−μsτ)−μss3,

where the definition, biological meaning, and values of each of parameter (values taken from [14]) are described in Table 1.

A brief description of the system (1) should clarify their meaning.

Let *S* be the total number of sandflies. The number of bites inflicted in the human host population in an infinitesimal time interval *dt* is *a*_*h*_ *S* *dt*, where *a*_*h*_ is the biting rate on humans. The number of bites inflicted by infected flies is *a*_*h*_ *S* *dt* *S*_3_/*S* = *a*_*h*_ *S* *dt* *s*_3_, where *S*_3_ is the number of infected flies.

Let now *X*_*h*_ be the total number of susceptible individuals in the human population. In an infinitesimal time interval *dt*, *X*_*h*_ varies as follows:

- a fraction of the infected bites are on uninfected individuals: *a*_*h*_ *S* *dt* *s*_3_ *x*_*h*_; a fraction *b* of these becomes latent, so *X*_*h*_ diminishes by *ba*_*h*_ *S* *dt* *s*_3_ *x*_*h*_;
- in the same time interval *r*_*h*_ *L*_*h*_ *dt* + *γ*_*h*_ *Z*_*h*_ individuals, latent and immune, revert to the susceptible condition, and *μ*_*h*_ *X*_*h*_ *dt* die by natural causes other than the disease;
- we must add an entrance term, due to natality, which we choose to be *α*_*h*_ *Y*_*h*_ *dt* + *μ*_*h*_ *N*_*h*_ *dt*, where *α*_*h*_ is the disease-induced mortality rate, *Y*_*h*_ is the number of infected humans, and *N*_*h*_ is the total number of humans needed to maintain a constant population.

Therefore we have

(2) dXh=αhYhdt+μhNh−bahSdts3xh+(dhLhdt+γhZhdt)−μhXhdt.

Dividing this equation by *N*_*h*_ *dt* and calling *S*/*N*_*h*_ = *m*_*h*_, we get the first equation of the system (1).

Equations indexed with *d* in the system (1) are related to the dog's subpopulation. They differ from the original model by Burattini et al. [14] by including control terms. Therefore, the term *ω* in the fifth and seventh equations refers to the treatment rate, according to which infected dogs are removed from the infectious compartment and are transferred to the susceptible one; the term *θ* in the fifth and sixth equations refers to the insecticide-impregnated collars, which by repelling the vector reduce the biting rate to the dogs; and finally the term *ξ* in the sixth and seventh equations refers to the culling of latent and infected dogs.

The last three equations of the system (1) refer to the flies. When infected, a fly remains in a latent stage for a period of time *τ*. This time corresponds to the extrinsic incubation period of the parasite inside the vector fly. Numerically it lasts for about half the life expectancy of the flies.

Let *S*_1_ be the number of susceptible flies. In an infinitesimal period of time *dt*, (*a*_*d*_((*Y*_*d*_ + *L*_*d*_)/*N*_*d*_)*dt*)*S*_1_ bites due to uninfected flies occur on latent and infected dogs (humans are not considered to be infective for flies; see [1]). A fraction *c* of the flies becomes latently infected as a result. Therefore, we have

(3) $$\begin{array}{c} dS_{1}=\mu_{s}\left(S_{2}+S_{3}\right)dt-\left(\frac{c}{\nu }\right)\left(\left(\frac{a_{d}}{\theta }\right)\left(y_{d}+l_{d}\right)\right)S_{a}dt, \end{array}$$

where the term *ν* refers to the transmission-blocking vaccine, which works by the action of specific antibodies that block the process of the parasite maturation inside the vector [9], thereby reducing the infectiousness of the vector and term *θ* as above.

Dividing by *S* = *S*_1_ + *S*_2_ + *S*_3_ and by *dt* we get the first of the last three equations of system (1).

## 3. Results

To simulate five possible control strategies, namely, dog treatment, insecticide-impregnated collars, culling, transmission-blocking vaccine, and vector control, several parameters were added to the original model by Burattini et al. [14]. We therefore simulated the system (1) with the parameters described in Table 1, to analyze the comparative effect of each of the five control strategies, being included one by one in the model. The range of the parameters related to control was assumed to vary from 1 (without control) to 1,000.

Dog treatment with chemotherapy based on meglumine antimoniate and allopurinol is the current standard to control leishmaniasis [17, 18]. In the model this strategy is simulated by varying the rate *ω* in the fifth and seventh equations, such that infected dogs are removed from the infectious compartment and are transferred to the susceptible compartment. 

The use of deltamethrin-impregnated dog collars [8] was simulated by varying the term *θ* in the fifth and sixth equations, based on the mechanism of repelling the vector and reducing the biting rate to the dogs.

Dog culling was simulated by varying the term *ξ* in the sixth and seventh equations, which represent an additional mortality rate to the natural mortality rate *μ*_*d*_ of dogs.

Transmission-blocking vaccines [9] act by raising antibodies in protected dogs, which impede the biding of leishmania to the sandflies midguts to curtail the transmission of the infection [19]. This strategy was simulated by varying the term *ν* in the last three equations of the system (1).

Finally, vector control, normally carried out by house spraying of insecticides [7], was simulated by simply increasing the natural mortality rate of sandflies *μ*_*s*_.

**Figure 1:**
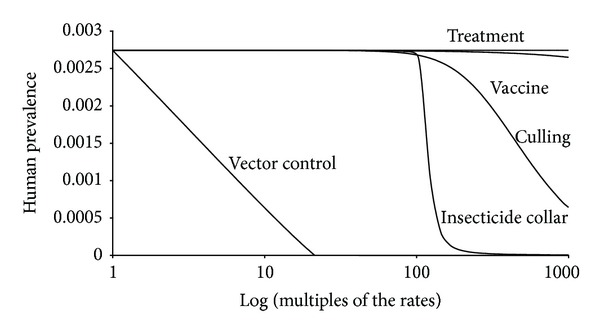
Result of the simulations of the system (1) with the parameters from Table 1.

The results of the simulations described previously can be seen in Figure 1.

Note that the effectiveness of the simulated control strategies ranges from practically in the case of dog treatment to complete null in the case of vector control. Treating dogs is practically ineffective in reducing disease in humans, followed by vaccine, which has a very small impact on human prevalence. Culling dogs, the official strategy in Brazil, is the next least effective, but it is necessary to increase the natural mortality rate of dogs by one thousand times to reduce human prevalence significantly. The use of insecticide-impregnated collars is very effective in controlling the disease in humans. However, as previously discussed and demonstrated in other studies, vector control is by far the most effective strategy in controlling ZVL in humans, being more than 50 times effective than culling dogs (see Figure 1).

In the context of the present model, the central parameter related to control is the force of infection, also known as the incidence-density rate. This parameter is defined as the per capita number of new infections per unit time, and it is normally denoted by the Greek letter *λ* [20]. For a vector-borne infection, *λ* is defined as [20]

(4) $$\begin{array}{c} \lambda \left(t\right)=m_{i}a_{i}b_{i}S_{3}\left(t\right), \end{array}$$

where *i* = *d* for dogs and *i* = *h* for humans and the components are the same as above. From the force of infection we can calculate the risk *R* of acquiring the infection as [21]

(5) $$\begin{array}{c} R=1-\exp\left[-\int_{0}^{\infty }\lambda \left(t\right)dt\right]=1-\exp\left[-m_{i}a_{i}b_{i}\int_{0}^{\infty }S_{3}\left(t\right)dt\right]. \end{array}$$

It is now possible to interpret our results. Each of the tested control strategies acts on one or more of the components of (5). Therefore, treating dogs should reduce the number of infected flies; culling should reduce the number of available dogs, which would theoretically reduce the number of available dogs in the transmission chain; the transmission-blocking vaccine was assumed to interfere with the probability of infection to the flies, reducing the total number of infected vectors; insecticide-impregnated collars reduce the biting rate, which is directly related with the risk of infection; and vector control directly reduces the number of infected flies.

## 4. Discussion

Ethical, legal and societal value issues related to animals should determine the way we handle them [22]. Unfortunately, some people and governments think that questions of humanity and justice have nothing to do with our treatment of animals [22]. As mentioned by Singer [23], “once we recognize that nonhuman animals have complex emotional and social needs, we begin to see animal abuse where others might not see it.” 

In ecological studies, it has been demonstrated that lethal methods of controlling wildlife may present difficulties in terms of conservation, ethics, welfare, and efficacy. It is therefore imperative to develop nonlethal alternatives and the advantages they might bring [24]. Concerns over widespread culling of wildlife have led to increasing interest in nonlethal control methods that target problem animals in particular [25]. The same arguments hold for domestic animals, in particular for such species like pets, which are increasingly being considered as part of the family. Therefore, alternative methods of controlling zoonosis transmitted by dogs other than simple destruction are, among other considerations, a matter of ethics. In addition, veterinarians in charge of decisions regarding implementation of culling actions find themselves in conflict between the officially required disease control policy and a public that is increasingly critical. Those veterinarians are faced with the challenge to defend the relevant decisions against all stakeholders and also themselves [26]. This brings additional ethical considerations to the culling problem.

In a quite interesting paper, Cohen et al. [27] present a model for describing people's fundamental moral attitudes in judgment of the culling animals in the context of animal disease. The authors defend that those fundamental moral attitudes are a two-layered concept. The first layer consists of deeply felt convictions about animals. The second layer consists of convictions derived from the first layer to serve as arguments in a debate on animal issues. These authors conclude that the value of an animal's life conviction was indeed the core argument against the culling of animals. 

Our model demonstrates that some alternative control methods of ZVL are not only feasible but also more effective that the culling of seropositive dogs. Two of the tested control strategies fared better than dog destruction, the use of insecticide-impregnated collars, and vector control. The latter has already been proposed in the context of other vector-borne infections [28–30], and, in spite of its logistical difficulties, vector control is the strategy with the highest impact on the infection's basic reproduction ratio [31, 32]. However, as mentioned previously, the central parameter related to control is the force of infection, and the analysis of the latter allows us to interpret our results in a straightforward way.

## 5. Conclusions

In conclusion, our model demonstrated that at least two alternative control strategies to culling dogs are more effective in reducing human prevalence of ZVL than the destruction of seropositive dogs, insecticide-impregnated collars, and vector control. It should be expected that a combination of those two strategies would have a more significant impact on human prevalence. Invariably we will not be able to put a collar on every dog or kill all the sandflies, but through a combination of both we could effectively reach the same result without needing to be as thorough with one method. Therefore, the traditional method of controlling ZVL, the destruction of seropositive dogs, can be abandoned without any harm to the human population.

Finally, although the treatment of infected dogs is practically ineffective in reducing human prevalence of ZVL, it should be provided on ethical and humanitarian grounds.

As mentioned by Singer P et al. [23] “universal common morality only supplies the core moral concepts and principles on the basis of which we can and should reflect on the appropriate treatment of animals.” 

## Figures and Tables

**Table 1:** Model's parameters. Values from Burattini et al. [14]. The indexes *h, d, * and *s* stand for humans, dogs, and sandflies, respectively.

Parameter	Biological meaning	Values
μ_*h*_	Natural mortality rate	4.11 × 10^−5^ days^−1^
α_*h*_	Disease-induced lethality	5.48 × 10^−3^ days^−1^
*a*_*h*_	Average daily biting rate	1.25 × 10^−1^ days^−1^
*m*_*h*_	Vector density per host	1.00 × 10^2^
*r*_*h*_	Spontaneous recovery rate	5.48 × 10^−4^ days^−1^
γ_*h*_	Loss of immunity rate	5.48 × 10^−4^ days^−1^
δ_*h*_	Latent recovery rate	1.10 × 10^−2^ days^−1^
φ_*h*_	Inverse of incubation period	4.00 × 10^−4^ days^−1^
σ_*h*_	Recovery rate of immunes	1.81 × 10^−3^ days^−1^
*b*_*h*_	Proportion of infective bites	1.00 × 10^−2^
μ_*d*_	Natural mortality rate	1.81 × 10^−4^ days^−1^
α_*d*_	Disease-induced lethality	1.81 × 10^−3^ days^−1^
*a*_*d*_	Average daily biting rate	1.25 × 10^−1^ days^−1^
*m*_*d*_	Vector density per host	5.00 × 10^2^
*r*_*d*_	Spontaneous recovery rate	2.74 × 10^−4^ days^−1^
γ_*d*_	Loss of immunity rate	2.74 × 10^−3^ days^−1^
ω_*d*_	Treatment rate	Variable
ν	Vaccination	Variable
ξ	Culling rate	Variable
θ	Insecticide collar	Variable
δ_*d*_	Latent recovery rate	8.22 × 10^−3^ days^−1^
φ_*d*_	Inverse of incubation period	1.10 × 10^−3^ days^−1^
σ_*d*_	Recovery rate of immunes	9.04 × 10^−4^ days^−1^
*b*_*d*_	Proportion of infective bites	2.00 × 10^−2^
μ_*s*_	Natural mortality rate	6.00 × 10^−2^ days^−1^
τ	Extrinsic incubation period	7.00 days
*c*	Proportion of infective bites	5.00 × 10^−2^

## References

[1] Tesh RB (1995). Control of zoonotic visceral leishmaniasis: is it time to change strategies?. *The American Journal of Tropical Medicine and Hygiene*.

[2] Quinnell RJ, Dye C (1994). Correlates of the peridomestic abundance of *Lutzomyia longipalpis* (Diptera: Psychodidae) in Amazonian Brazil. *Medical and Veterinary Entomology*.

[3] Quinnell RJ, Courtenay O, Garcez L, Dye C (1997). The epidemiology of canine leishmaniasis: transmission rates estimated from a cohort study in Amazonian Brazil. *Parasitology*.

[4] Falqueto A, Ferreira AL, Dos Santos CB (2009). Cross-sectional and longitudinal epidemiologic surveys of human and canine *Leishmania infantum* visceral infections in an endemic rural area of Southeast Brazil. *The American Journal of Tropical Medicine and Hygiene*.

[5] Courtenay O, Quinnell RJ, Garcez LM, Shaw JJ, Dye C (2002). Infectiousness in a cohort of Brazilian dogs: why culling fails to control visceral leishmaniasis in areas of high transmission. *Journal of Infectious Diseases*.

[6] Ashford DA, David JR, Freire M (1998). Studies on control of visceral leishmaniasis: impact of dog control on canine and human visceral leishmaniasis in Jacobina, bahia, Brazil. *The American Journal of Tropical Medicine and Hygiene*.

[7] WHO Expert Committee (1990). Control of the leishmaniases. *World Health Organization Technical Report Series*.

[8] Reithinger R, Coleman PG, Alexander B, Vieira EP, Assis G, Davies CR (2004). Are insecticide-impregnated dog collars a feasible alternative to dog culling as a strategy for controlling canine visceral leishmaniasis in Brazil?. *International Journal for Parasitology*.

[9] Palatnik-de-Sousa CB, Silva-Antunes I, Morgado ADA, Menz I, Palatnik M, Lavor C (2009). Decrease of the incidence of human and canine visceral leishmaniasis after dog vaccination with Leishmune in Brazilian endemic areas. *Vaccine*.

[10] Dye C (1996). The logic of visceral leishmaniasis control. *The American Journal of Tropical Medicine and Hygiene*.

[11] Vieira JB, Coelho GE (1998). Visceral leishmaniasis or kala-azar: the epidemiological and control aspects. *Revista da Sociedade Brasileira de Medicina Tropical*.

[12] Barreto AVP (2010). Northeastern symposium on leishmaniasis. *Clinica Veterinária*.

[13] Singer P (2003). *Animal Liberation*.

[14] Burattini MN, Coutinho FAB, Lopez LF, Massad E (1998). Modelling the dynamics of leishmaniasis considering human, animal host and vector populations. *Journal of Biological Systems*.

[15] Braga MD, Coêlho IC, Pompeu MM (1998). Control of canine visceral leishmaniasis: comparison of results from a rapid elimination program of serum-reactive dogs using an immunoenzyme assay and slower elimination of serum-reactive dogs using filter paper elution indirect immunofluorescence. *Revista da Sociedade Brasileira de Medicina Tropical*.

[16] Paranhos-Silva M, Nascimento EG, Melro MCBF (1998). Cohort study on canine emigration and Leishmania infection in an endemic area for American visceral leishmaniasis. Implications for the disease control. *Acta Tropica*.

[17] João A, Pereira MA, Cortes S, Santos-Gomes GM (2006). Canine leishmaniasis chemotherapy: dog's clinical condition and risk of Leishmania transmission. *Journal of Veterinary Medicine A*.

[18] Ikeda-Garcia FA, Lopes RS, Ciarlini PC (2007). Evaluation of renal and hepatic functions in dogs naturally infected by visceral leishmaniasis submitted to treatment with meglumine antimoniate. *Research in Veterinary Science*.

[19] Saraiva EM, Barbosa ADF, Santos FN (2006). The FML-vaccine (Leishmune) against canine visceral leishmaniasis: a transmission blocking vaccine. *Vaccine*.

[20] Massad E, Behrens RH, Burattini MN, Coutinho FAB (2009). Modeling the risk of malaria for travelers to areas with stable malaria transmission. *Malaria Journal*.

[21] Bailey NTJ (1975). *The Mathematical Theory of Infectious Diseases and Its Applications*.

[22] Beauchamp TL, Orlans FB, Dresser R, Morton DB, Gluck JP (2008). *The Human Use of Animals*.

[23] Singer P P, Macedo S, Ober J (2006). Morality, reason, and the rights of animals. *Primates and Philosophers: How Morality Evolved*.

[24] Baker SE, Ellwood SA, Watkins R, Macdonald DW (2005). Non-lethal control of wildlife: using chemical repellents as feeding deterrents for the European badger *Meles meles*. *Journal of Applied Ecology*.

[25] Shivik JA, Treves A, Callahan P (2003). Nonlethal techniques for managing predation: primary and secondary repellents. *Conservation Biology*.

[26] Hartnack S, Doherr MG, Grimm H, Kunzmann P (2009). Mass culling in the context of animal disease outbreaks. *Deutsche Tierarztliche Wochenschrift*.

[27] Cohen NE, Brom FWA, Stassen EN (2009). Fundamental moral attitudes to animals and their role in judgment: an empirical model to describe fundamental moral attitudes to animals and their role in judgment on the culling of healthy animals during an animal disease epidemic. *Journal of Agricultural and Environmental Ethics*.

[28] Massad E, Coutinho FAB, Burattini MN, Lopez LF (2001). The risk of yellow fever in a dengue-infested area. *Transactions of the Royal Society of Tropical Medicine and Hygiene*.

[29] Lopez LF, Coutinho FAB, Burattini MN, Massad E (2002). Threshold conditions for infection persistence in complex host-vectors interactions. *Comptes Rendus—Biologies*.

[30] Coutinho FAB, Burattini MN, Lopez LF, Massad E (2006). Threshold conditions for a non-autonomous epidemic system describing the population dynamics of dengue. *Bulletin of Mathematical Biology*.

[31] Marques CA, Forattini OP, Massad E (1994). The basic reproduction number for dengue fever in Sao Paulo State, Brazil: 1990-1991 epidemic. *Transactions of the Royal Society of Tropical Medicine and Hygiene*.

[32] Massad E, Coutinho FAB, Yang HM, de Carvalho HB, Mesquita F, Burattini MN (1994). The basic reproduction ratio of HIV among intravenous drug users. *Mathematical Biosciences*.

